# Synthesis, Leishmanicidal and Cytotoxic Activity of Triclosan-Chalcone, Triclosan-Chromone and Triclosan-Coumarin Hybrids 

**DOI:** 10.3390/molecules190913251

**Published:** 2014-08-28

**Authors:** Elver Otero, Sebastián Vergara, Sara M. Robledo, Wilson Cardona, Miguel Carda, Ivan D. Vélez, Carlos Rojas, Felipe Otálvaro

**Affiliations:** 1 Química de Plantas Colombianas, Instituto de Química, Facultad de Ciencias Exactas y Naturales, Universidad de Antioquia UdeA, Calle 70 No. 52-21, A.A 1226 Medellín, Colombia; E-Mails: elverluisotero@yahoo.es (E.O.); sebitasnano@hotmail.com (S.V.); 2 PECET, Instituto de Investigaciones Médicas, Facultad de Medicina, Universidad de Antioquia UdeA, Calle 62 No. 52-59, Lab 632, A.A 1226 Medellín, Colombia; E-Mails: sara.robledo@udea.edu.co (S.M.R.); ivan.velez@udea.edu.co (I.D.V.); 3 CIDEPRO-Center for Development of Products against Tropical Diseases, Carrera 53 No 61-30, Sede de Investigacion Universitaria, A.A 1226 Medellin, Colombia; 4 Departamento de Química Inorgánica y Orgánica, Universidad Jaume I, E-12071 Castellón, Spain; E-Mail: mcarda@qio.uji.es; 5 SIN-BIO-ME-NA, Facultad de Ciencias Exactas y Naturales, Universidad de Antioquia UdeA, Calle 70 No. 52-21, A.A 1226 Medellín, Colombia; E-Mails: pipelion@quimica.udea.edu.co (F.O.); kimikrlos@hotmail.com (C.R.)

**Keywords:** leishmaniasis, antiprotozoal, triclosan, coumarin, chromone, chalcone, hybrids

## Abstract

Twelve hybrids derived from triclosan were obtained via Williamson etherification of O-triclosan alkyl bromide plus chalcone and O-coumarin or O-chromone alkyl bromide plus triclosan, respectively. Structures of the products were elucidated by spectroscopic analysis. The synthesized compounds were evaluated for antileishmanial activity against *L. (V) panamensis* amastigotes. Cytotoxic activity was also evaluated against mammalian U-937 cells. Compounds **7**–**9** and **17**, were active against *Leishmania* parasites (EC_50_ = 9.4; 10.2; 13.5 and 27.5 µg/mL, respectively) and showed no toxicity toward mammalian cells (>200 µg/mL). They are potential candidates for antileishmanial drug development. Compounds **25**–**27**, were active and cytotoxic. Further studies using other cell types are needed in order to discriminate whether the toxicity shown by these compounds is against tumor or non-tumor cells. The results indicate that compounds containing small alkyl chains show better selectivity indices. Moreover, Michael acceptor moieties may modify both the leishmanicidal activity and cytotoxicity. Further studies are required to evaluate if the *in vitro* activity against *Leishmania panamensis* demonstrated here is also observed *in vivo*.

## 1. Introduction

Leishmaniasis is a group of diseases caused by protozoan parasites of the genus *Leishmania*, which infect and replicate inside macrophages of the vertebrate host. The diseases is a major health problem because is present in 98 countries and three territories worldwide, affecting mostly low-income people in rural areas of tropical and subtropical countries. Approximately 0.7 to 1.2 million cutaneous leishmaniasis (CL) cases occur annually. Afghanistan, Algeria, Colombia, Brazil, Iran, Syria, Ethiopia, North Sudan, Costa Rica and Peru, together account for 70% to 75% of global estimated CL incidence [1]. *Leishmania (Viannia) panamensis* is one of the most important causal agent of CL in Central and South America [2]. The different forms of leishmaniasis require expensive treatments, and the currently used medicines, pentavalent antimonials, pentamidine isothianate and miltefosine, show high toxicity and therefore severe side effects and therefore there is an urgent need for new drugs [3]. However, due to the lack of interest shown by the pharmaceutical industry to develop drugs against neglected diseases, it is necessary to join forces to develop new and better drugs to manage the disease and help patients to improve their quality of life [4].

Triclosan is an uncompetitive inhibitor of purified enoyl-acyl carrier protein reductase (ENR), which has demonstrated inhibitory activity *in vitro* against *Plasmodium falciparum* [5,6,7,8]. A previous study showed that triclosan and quinoline-triclosan hybrids with shorter spacers, that is three and five methylene units, have *in vitro* activity against axenic and intracellular amastigotes with effective concentration (EC_50_) below of 24 µg/mL) of *Leishmania panamensis* [9]. Similarly, antileishmanial activity of several chalcones has been reported [10,11,12]. The most promising of this class of compounds is licochalcone A, an oxygenated chalcone isolated from the roots of the Chinese plant *Glycyrrhiza* spp., which inhibits the fumarate reductase, a selective target present in the mitochondria of the parasite [13].

Coumarins and chromones are important classes of compounds having versatile biological activities [14,15,16,17,18,19,20,21,22]. Both moieties are well known for their antiprotozoal activity. Some synthetic chromones were effective against *L. donovani* [23] and *L. major* [24] in *in vivo* studies. Aurapten, a 7-geranyloxycoumarin, was extracted from the Rutaceae species *Esenbeckia febrifuga*. This compound shows significant growth inhibition with a 50% inhibitory concentration (IC_50_) of 30 µM against *L. major* [25]. Three coumarins isolated from the leaves of *Galipea panamensis* were tested against axenic amastigote forms of *L. panamensis* and displayed 50% effective concentrations (EC_50_) of 9.9, 10.5, and 14.1 µg/mL, respectively [26]. In addition, several 4-arylcoumarins were found to strongly inhibit the protozoan parasites of *L. donovani*, particularly 4-(3,4-dimethoxyphenyl)-6,7-dimethoxy-coumarin that exhibits potent activity on intracellular amastigotes with a selectivity index (SI) of 265, twice that shown by amphotericin B (SI = 140) [27].

The combination of two pharmacological agents into a single molecule, called hybrid molecule, is an emerging strategy in medicinal chemistry and drug discovery research [28,29]. These hybrid molecules may display dual activity but do not necessarily act on the same biological target [30,31,32,33,34].

**Scheme 1:**
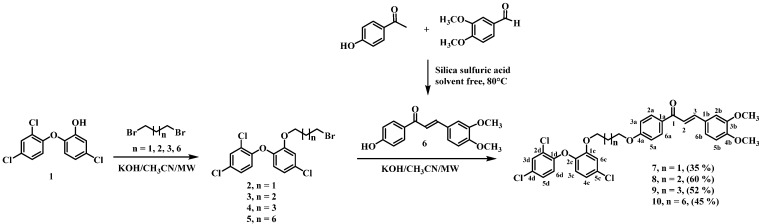
Synthetic pathway to triclosan-chalcone hybrids.

**Scheme 2:**
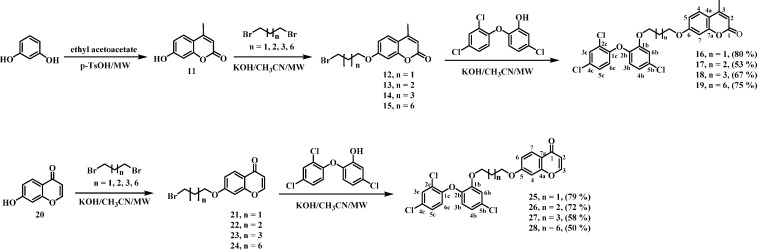
Synthetic pathway to triclosan-coumarin and triclosan-chromone hybrids.

In this work, four chalcone-triclosan, chromone-triclosan, and coumarin-triclosan hybrids (Scheme 1 and Scheme 2) were synthesized and their cytotoxic and leishmanicidal activities determined in the search for new therapeutic alternatives for the treatment of leishmaniasis.

## 2. Results and Discussion

### 2.1. Chemistry

Triclosan-chalcone hybrids **7**–**10** were obtained via microwave assisted Williamson etherification [35] between bromoalkyltriclosan **2**–**5** and 3,4-dimethoxy-4'-hydroxychalcone (**6**). Reaction yields ranged between 35% and 60%. Chalcone **6** was prepared using a previously described method [36] (Scheme 1). 

Triclosan-coumarin and triclosan-chromone hybrids were obtained following the same synthetic strategy (Scheme 2). Initially, 7-hydroxy-4-methylcoumarin (**11**, obtained by microwave assisted Pechmann reaction between resorcinol and ethyl acetoacetate [37]) or commercially available 7-hydroxychromone (**20**) were treated with potassium hydroxide and 1,ω-dibromoalkanes (ω = 3, 4, 5, 8) to obtain the respective bromoalkyl derivatives in yields similar to previous reports [9,38,39] but in significantly shorter times. These compounds were coupled with triclosan to produce compounds **16**–**19** and **25**–**28** in 50%–80% yields. Remarkably, low yields were obtained when bromoalkyltriclosan analogs were used as tactical variants.

### 2.2. Antileishmanial and Cytotoxic Activities

The leishmanicidal activity and cytotoxicity of the synthesized compounds was evaluated following previously reported method [40,41]. Leishmanicidal activity was reported as percentage of inhibition at 20 µg/mL and 50% effective concentration (EC_50_) values. On the other hand, cytotoxicity was reported as 50% lethal concentration (LC_50_) values. Results are summarized in Table 1.

**Table 1:** *In vitro* toxicity and anti-Leishmania activity of triclosan-chalcone, triclosan-chromone and triclosan-coumarin hybrids.

Compound	Cytotoxicity	Leishmanicidal activity		SI ^d^
	LC_50_ (μg/mL, µM) ^a^	% Inhibition ^b^	EC_50_ (μg/mL, µM) ^c^	
**7**	>200.0, >326.7	67.4 ± 17.2	9.4 ± 1.3, 15.4	>21.3
**8**	>200.0, >319.4	67.8 ± 19.0	10.2 ± 1.8, 16.3	>19.6
**9**	>200.0, >311,4	69.5 ± 8.6	13.5 ± 3.6, 21.1	>14.8
**10**	>200.0, >293.2	23.3 ± 5.2	NE ^e^	NC ^f^
**16**	>200.0, >396.8	34.0 ± 0.3	NE ^e^	NC ^f^
**17**	>200.0, >386.1	57.1 ± 11.5	27.5 ± 0.8, 53.1	>7.3
**18**	>200.0, >375.9	0.0	NE ^e^	NC ^f^
**19**	>200.0, >348.4	0.0	NE ^e^	NC ^f^
**25**	6.4 ± 0.8, 13.1	94.4 ± 2.9	2.7 ± 0.4, 5.5	2.4
**26**	15.8 ± 4.3, 31.3	91.0 ± 9.6	7.5 ± 0.2, 14.9	2.1
**27**	25.8 ± 4.2, 49.8	75.5 ± 1.5	16.0 ± 1.0, 30.9	1.6
**28**	80.0 ± 18.5, 142.8	28.4 ± 2.1	NE ^e^	NC ^f^
Triclosan	22.1 ± 3.1, 76.3	61.8 ± 5.5	18.3 ± 2.01, 63.2	1.3
3,4-Dimethoxy-4'-hydroxychalcone ( **6**)	13.9 ± 1.4, 48.9	52.4 ± 6.5	20.03 ± 1.4, 70.5	0.7
7-Hydroxychromone ( **20**)	>200.0, >1234.6	14.8 ± 0.9	NE ^e^	NC ^f^
7-Hydroxy-4-methylcoumarin ( **11**)	98.2 ± 6.7, 557.4	15.3 ± 0.1	NE ^e^	NC ^f^
*Sb(V)* ^g^	495.9 + 55.6	79.4 ± 2.1 ^h^	6.3 + 0.9	78.7
*Amphotericin B*	42.1 ± 2.0, 45.6	69.1 ± 1.3 ^i^	0.06 ± 0.01, 0.1	592

± Standard deviation; ^a^ LC_50_: Lethal Concentration 50; ^b^ % Inhibition at 20 µg/mL; ^c^ EC_50_: Effective Concentration 50; ^d^ IS: Selectivity Index: = LC_50_/EC_50_; ^e^ NE: Not evaluated because inhibition was below 50% at 20 µg/mL; ^f^ NC: Not calculated because EC_50_ wasn’t determined; ^g^ SbV: pentavalent antimonial meglumine antimoniate; ^h^ Dose employed: 10 µg/mL; ^i^ Dose employed: 0.05 µg/mL.

According to the results shown in Table 1, triclosan, chalcone and compounds **7**–**9**, **17** and **25**–**27** showed activity against intracellular amastigotes of *L. (V) panamensis* with more than 50% inhibition at 20 µg/mL. The most active compounds were **25**–**27**, with inhibitions of 94.4%, 91.0% and 75.5%, respectively. Compounds **7**–**9** and **17** showed a moderate activity with inhibitions of 67.4%, 67.8%, 69.5% and 57.1%, respectively. On the other hand, high toxicity was measured for compounds **25**–**27**, with LC_50_ < 26 µg/mL. Lower toxic activity was obtained for compounds **7**–**9** and **17** (LC_50_ > 200.0 µg/mL). Weak to negligible leishmanicidal activity and no toxicity were detected for compounds **10**, **16**, **18**, and **19** (inhibition <50% at 20 µg/mL and LC_50_ higher than 200 µg/mL, respectively). Compound **28** displayed very low activity and moderate cytotoxicity with values of 28.4% and 80.0 µg/mL, respectively.

The effective concentration against the intracellular forms of *L. (V) panamensis* was also measured for those compounds showing percentages of inhibition higher than 50% at 20 µg/mL (Table 1). Thus, compounds **10**, **16**, **18**, **19** and **28** were not evaluated. The most active compounds for intracellular parasites were **25**, **26** and **7** with EC_50_ of 2.7, 7.5 and 9.4 µg/mL, respectively, followed by compounds **8**, **9** and **27** with EC_50_ values of 10.2, 13.5 and 16.0 µg/mL, respectively (Table 1). All compounds showed leishmanicidal activity higher than cytotoxicity and therefore, selectivity indexes higher than 1. The best SI´s were observed for compounds **7**–**9** and **17** with values higher than of 21.3, 19.6, 14.8 and 7.3, respectively. Although compound **25** showed better activity than meglumine antimoniate, its SI is affected by the high cytotoxicity.

On a structure-activity relationship basis, it is interesting to note the synergistic effect of the parent subunits in the hybrids in comparison with the unlinked cases. For example, chalcone 6 and triclosan are less potent and more cytotoxic individually than their hybrids **7**–**9**. This phenomenon can also be observed in compounds **25**–**27** in which an increase in activity is evident in the hybrids at the expense of cytotoxicity compared to the individual units. The length of the alkyl linker also plays a pivotal role, with an inverse correlation between increased length and activity for the evaluated cases.

One possible mechanisms of action for these compounds may be formulated in terms of conjugated addition of nucleophilic amino acid residues present in biomolecules of the natural receptors, in a Michael type mechanism. This mechanism has been reported for other α,β-unsaturated compounds such as lactones and cinnamic acid esters [42,43,44]. In this regard, the low reactivity shown by triclosan-coumarin hybrids could be rationalized in terms of steric hindrance and electronic deactivation caused by the methyl group at the β-position.

## 3. Experimental Section

### 3.1. Chemical Synthesis

#### 3.1.1. General Remarks

The syntheses were carried out in a MW domestic oven adapted for the use of a reflux condenser and magnetic stirrer, at constant power (400 W). NMR spectra were recorded as CDCl_3_ solutions on an AMX 300 instrument (Bruker, Billerica, MA, USA) operating at 300 MHz for 1H and 75 MHz for ^1^C. Chemical shifts (δ) are expressed in ppm with the solvent peak as reference and TMS as an internal standard; coupling constants (J) are given in Hertz (Hz). High resolution mass spectra were recorded using electrospray ionization mass spectrometry (ESI-MS). A QTOF Premier instrument with an orthogonal Z-spray-electrospray interface (Waters, Manchester, UK) was used operating in the W-mode. The drying and cone gas was nitrogen set to flow rates of 300 and 30 L/h, respectively. Methanol sample solutions (*ca.* 1 × 10^−5^ M) were directly introduced to the ESI spectrometer at a flow rate of 10 µL/min. A capillary voltage of 3.5 kV was used in the positive scan mode, and the cone voltage set to *U*c = 10 V. For the accurate mass measurements, a 2 mg/L standard solution of leucine enkephalin was introduced via the lock spray needle at a cone voltage set to 85 V and a flow rate of 30 μL/min. IR spectra were recorded on a Spectrum RX I FT-IR system (Perkin-Elmer, Waltham, MA, USA) in KBr disks. Elemental analysis were recorded on TruSpec Micro Series equipment (LECO Corporation, St. Joseph, MI, USA). Commercially available reagents were used as received. Silica gel 60 (0.063–0.200 mesh, Merck, Whitehouse Station, NJ, USA) was used for column chromatography, and precoated silica gel plates (Merck 60 F254 0.2 mm) were used for thin layer chromatography (TLC).

#### 3.1.2. General Procedure for the Synthesis of Bromoalkyl Derivatives

Triclosan, chromone or coumarin (1 mmol, 1 eq.), potassium hydroxide (3 eq.) and acetonitrile (10 mL), were placed in a 50 mL flat-bottomed flask equipped with a magnetic stirring bar. The mixture was stirred and heated to reflux for a period of 5 min, under microwave irradiation. Then, 1,ω-dibromoalkane (1.1 eq.) was added to the reaction mixture which was refluxed for 30 min. The crude reaction mixture was concentrated on a rotatory evaporator, and the residue was purified by column chromatography over silica gel eluting with hexane and a mixture of hexane-ethyl acetate (9:1 ratio) to obtain the bromoalkyl derivatives in yields ranging between 60%–85%. Monitoring of the reaction progress and product purification was carried out by TLC.

*7-[(8-Bromooctyl)oxy]-4-methyl-2H-chromen-2-one* (**15**). Yield 60% (1.02 mmol, 375.3 mg); pale yellow oil; IR (cm^−1^): ν_max_ 2933 (C-H), 1737 (C=O), 1612 (C=C), 1463 (C=C_Ar_), 1261 (C-O-C), 1199 ((C=O)-O), 842 (C-H_Ar_); ^1^H-NMR (CDCl_3_): δ 1.35–1.46 (4H, m), 1.46–1.58 (4H, m), 1.78–1.97 (4H, m), 2.43 (S, CH_3_), 3.45 (CH_2_Br, t, *J* = 6.9 Hz), 4.05 (CH_2_O-, t, *J* = 6.6 Hz), 6.16 (H2, s), 6.83 (H_7_, d, *J* = 2.4 Hz), 6.88 (H_5_, dd, *J* = 8.8, 2.4 Hz), 7.52 (H_4_, d, *J* = 8.8 Hz); ^13^C-NMR (CDCl_3_): δ 18.67 (CH_3_), 25.88 (CH_2_), 28.08 (CH_2_), 28.66 (CH_2_), 28.96 (CH_2_), 29.14 (CH_2_), 32.77 (CH_2_), 33.95 (CH_2_Br), 68.65 (CH_2_O), 101.37 (C_7_), 111.84 (C_5_), 112.68 (C_2_), 113.45 (C_4a_), 125.49 (C_4_), 152.61 (C_7a_), 155.33 (C_3_), 161.38 (C_6_), 162.23 (C=O).

*7-(4-Bromobutoxy)-4H-chromen-4-one* (**22**). Yield 82% (1.52 mmol, 450.78 mg); pale yellow oil; IR (cm^−1^): ν_max_ 2953 (C-H), 1639 (C=O), 1595 (C=C), 1438 (C=C_Ar_), 1265 (C-O-C), 856 (C-H_Ar_); ^1^H-NMR (CDCl_3_): δ 1.98–2.09 (2H, m), 2.09–2.18 (2H, m), 3.53 (CH_2_Br, t, *J* = 6.5 Hz), 4.12 (CH_2_O, t, *J* = 6.0 Hz), 6.31 (H_2_, d, *J* = 6.1 Hz), 6.86 (H_4_, d, *J* = 2.3 Hz), 6.99 (H_6_, dd, *J* = 9.0, 2.2 Hz), 7.81 (H_3_, d, *J* = 6.0 Hz), 8.14 (H_7_, d, *J* = 9.0 Hz); ^13^C-NMR (CDCl_3_): δ 27.63 (CH_2_), 29.29 (CH_2_), 33.17 (CH_2_Br), 67.58 (CH_2_O), 100.93 (C_4_), 112.97 (C_6_), 114.75 (C_2_), 118.83 (C_7a_), 127.26 (C_7_), 154.88 (C_3_), 158.24 (C_4a_), 163.35 (C_5_), 177.02 (C=O).

*7-[(5-Bromopentyl)oxy]-4H-chromen-4-one* (**23**). Yield 85% (1.57 mmol, 489.3 mg); pale yellow oil; IR (cm^−1^): ν_max_ 2929 (C-H), 1649 (C=O), 1595 (C=C), 1439 (C=C_Ar_), 1267 (C-O-C), 856 (C-H_Ar_); ^1^H-NMR (CDCl_3_): δ 1.61–1.74 (2H, m), 1.83–1.93 (2H, m), 1.93–2.04 (2H, m), 3.48 (CH_2_Br, t, *J* = 6.7 Hz), 4.08 (CH_2_O, t, *J* = 6.3 Hz), 6.30 (H_2_, d, *J* = 6.1 Hz), 6.84 (H_4_, d, *J* = 2.3 Hz), 6.98 (H_6_, dd, *J* = 8.9, 2.3 Hz), 7.80 (H_3_, d, *J* = 6.0 Hz), 8.12 (H_7_, d, *J* = 9.0 Hz); ^13^C-NMR (CDCl_3_): δ 24.74 (CH_2_), 28.17 (CH_2_), 32.37 (CH_2_), 33.51 (CH_2_Br), 68.29 (CH_2_O), 100.88 (C_4_), 112.94 (C_6_), 114.81 (C_2_), 118.72 (C_7a_), 127.18 (C_7_), 154.89 (C_3_), 158.25 (C_4a_), 163.49 (C_5_), 177.05 (C=O).

*7-[(8-Bromooctyl)oxy]-4H-chromen-4-one* (**24**). Yield 71% (1.31 mmol, 464 mg); yellow oil; IR (cm^−1^): ν_max_ 2939 (C-H), 1653 (C=O), 1596 (C=C), 1444 (C=C_Ar_), 1227 (C-O-C), 856 (C-H_Ar_); ^1^H-NMR (CDCl_3_): δ 1.34–1.46 (4H, m), 1.46–1.60 (4H, m), 1.77–1.97 (4H, m), 3.45 (CH_2_Br, t, *J* = 6.8 Hz), 4.08 (CH_2_O, t, *J* = 6.5 Hz), 6.31 (H_2_, d, *J* = 6.1 Hz), 6.86 (H_4_, d, *J* = 2.3 Hz), 7.01 (H_6_, dd, *J* = 8.9, 2.3 Hz), 7.81 (H_3_, d, *J* = 6.0 Hz), 8.14 (H_7_, d, *J* = 8.9 Hz); ^13^C-NMR (CDCl_3_): δ 25.88 (CH_2_), 28.07 (CH_2_), 28.66 (CH_2_), 28.93 (CH_2_), 29.13 (CH_2_), 32.76 (CH_2_), 33.95 (CH_2_Br), 68.65 (CH_2_O), 100.88 (C_4_), 112.92 (C_6_), 114.88 (C_2_), 118.64 (C_7a_), 127.17 (C_7_), 154.86 (C_3_), 158.31 (C_4a_), 163.70 (C_5_), 177.11 (C=O).

#### 3.1.3. General Procedure for the Synthesis of Triclosan-Chalcone Hybrids

Chalcone (1.1 eq.), potassium hydroxide (2 eq.) and acetonitrile (10 mL), were placed in a 50 mL flat-bottomed flask equipped with a magnetic stirring bar. The mixture was stirred and heated to reflux for a period of 5 min, under microwave irradiation. Then, bromoalkyltriclosan (100 mg, 1 eq.) was added to the reaction mixture which was then refluxed for 30 min. The crude reaction mixture was concentrated on a rotatory evaporator, and the residue was purified by column chromatography over silica gel eluting with hexane-ethyl acetate (9:1 ratio) to obtain the triclosan-chalcone hybrids in yields between 35%–60%. Monitoring of the reaction progress and product purification was carried out by TLC.

*(2E)-1-(4-{3-[5-Chloro-2-(2,4-dichlorophenoxy)phenoxy]propoxy}phenyl)-3-(3,4-dimethoxyphenyl) prop-2-en-1-one* (**7**). Yield 35% (0.085 mmol, 52 mg); yellow solid, M.p. 129–130 °C; IR (cm^−1^): ν_max_ 2935 (C-H), 1601 (C=O), 1512 (C=C), 1471 (C=C_Ar_), 1265 (C-O-C), 1160 ((C=O)-O), 800 (C-H_Ar_), 742 (C–Cl); ^1^H-NMR (CDCl_3_): δ 2.13–2.25 (2H, m), 3.96 (CH_2_O, t, *J* = 6.1 Hz), 3.98 (OCH_3_, s), 4.01 (OCH_3_, s), 4.18 (CH_2_O, t, *J* = 5.8 Hz), 6.62 (H_3c_, d, *J* = 8.8 Hz), 6.90 (H_6d_, d, *J* = 8.8 Hz), 6.95 (H_3a_, H_5a_, d, *J* = 8.4 Hz), 6.99 (H_4c_, dd, *J* = 6.5, 2.5), 6.99–7.03 (H_5b_, H_6b_, m), 7.06 (H_6c_, d, *J* = 2.5 Hz), 7.21 (H_2b_, d, J = 1.8 Hz), 7.29 (H_5d_, dd, *J* = 8.5, 1.8 Hz), 7.45 (H_3d_, d, *J* = 2.5 Hz), 7.46 (H_2_, d, *J* = 15.5 Hz), 7.81 (H_3_, d, *J* = 15.5 Hz), 8.06 (H_2a_, H_6a_, d, *J* = 8.9 Hz); ^13^C-NMR (CDCl_3_): δ 28.91 (CH_2_), 56.0 (2OCH_3_), 63.85 (CH_2_O), 65.10 (CH_2_O), 110.10 (C_2b_), 111.20 (C_5b_), 114.20 (C_3a_, C_5a_), 114.70 (C_6c_), 117.40 (C_3c_), 119.90 (C_6b_), 121.30 (C_6d_), 122.2 (C_4c_), 122.60 (C_2_), 123.0 (C_2d_), 124.10 (C_1b_), 127.70 (C_5d_), 128.10 (C_5c_), 130.20 (C_4d_), 130.80 (C_2a_, C_6a_), 130.90 (C_1a_), 131.4 (C_3d_), 142.70 (C_3_), 144.20 (C_2c_), 149.30 (C_1c_), 150.80 (C_3b_), 151.30 (C_1d_), 152.60 (C_4b_), 162.40 (C_4a_), 188.82 (C=O); EIMS: *m/z* 613.0946 [M + H]^+^, for C_32_H_28_Cl_3_O_6_: 613.0951. Anal. Calc. for C_32_H_27_Cl_3_O_6_: C 62.61, H 4.43. Found C 63.15, H 4.72.

*(2E)-1-(4-{4-[5-Chloro-2-(2,4-dichlorophenoxy)phenoxy]butoxy}phenyl)-3-(3,4-dimethoxyphenyl) prop-2-en-1-one* (**8**). Yield 60% (0.142 mmol, 89.8 mg); yellow solid, M.p. 103–105 °C; IR (cm^−1^): ν_max_ 2954 (C-H), 1601 (C=O), 1512 (C=C), 1472 (C=C_Ar_), 1262 (C-O-C), 1163 ((C=O)-O), 800 (C-H_Ar_), 794 (C–Cl); ^1^H-NMR (CDCl_3_): δ 1.72–1.84 (2H, m), 1.84–1.97 (2H, m), 3.98 (OCH_3_, s), 4.01 (OCH_3_, s), 4.05 (2CH_2_O, t, *J* = 6.4 Hz), 6.68 (H_3c_, d, *J* = 8.8 Hz), 6.92–7.05 (H_6c_, H_3a_, H_5a_, H_5b_, H_6b_, H_6d_), 7.12 (H_4c_, dd, *J* = 8.8, 2.5 Hz), 7.21 (H_2b_, d, *J* = 1.7), 7.28 (H_5d_, dd, *J* = 8.3, 1.6 Hz), 7.44 (H_3d_, d, *J* = 2.4 Hz), 7.45 (H_2_, d, *J* = 15.5 Hz), 7.81 (H_3_, d, *J* = 15.5 Hz), 8.06 (H_2a_, H_6a_, d, *J* = 8.7 Hz); ^13^C-NMR (CDCl_3_): δ 25.55 (CH_2_), 25.64 (CH_2_), 56.03 (2OCH_3_), 67.46 (CH_2_O), 68.58 (CH_2_O), 110.11 (C_2b_), 111.15 (C_5b_), 114.24 (C_3a_, C_5a_), 114.80 (C_6c_), 117.80 (C_3c_), 119.70 (C_6b_), 121.16 (C_6d_), 122.25 (C_4c_), 122.60 (C_2_), 123.0 (C_2d_), 124.35 (C_1b_), 127.61 (C_5c_), 127.83 (C_2c_), 128.12 (C_5d_), 130.17 (C_4d_), 130.7 (C_3d_), 130.76 (C_2a_, C_6a_), 131.24 (C_1a_), 143.01 (C_3_), 144.35 (C_2c_), 149.25 (C_1c_), 150.85 (C_3b_), 151.30 (C_1d_), 152.60 (C_4b_), 162.68 (C_4a_), 188.80 (C=O); EIMS: *m/z* 627.1111 [M + H]^+^, Calcd for C_33_H_30_Cl_3_O_6_: 627.1108. Anal. Calc. for C_33_H_29_Cl_3_O_6_: C 63.12, H 4.65. Found C 62.34, H 4.79.

*(2E)-1-[4-({5-[5-Chloro-2-(2,4-dichlorophenoxy)phenoxy]pentyl}oxy)phenyl]-3-(3,4-imethoxyphenyl) prop-2-en-1-one* (**9**). Yield 52% (0.119 mmol, 76 mg); yellow solid, M.p. 117–120 °C; IR (cm^−1^): ν_max_ 2949 (C-H), 1603 (C=O), 1510 (C=C), 1472 (C=C_Ar_), 1263 (C-O-C), 1159 ((C=O)-O), 862 (C-H_Ar_), 742 (C–Cl); ^1^H-NMR (CDCl_3_): δ 1.35–1.51 (2H, m), 1.69–1.86 (4H, m), 3.98 (OCH_3_, s), 4.01 (OCH_3_, s), 3.81–4.17 (4H, m), 6.66 (H_3c_, d, *J* = 8.8 Hz), 6.94 (H_4c_ , dd, *J* = 8.3, 1.4), 6.97–7.06 (H_3a_, H_5a_, H_6d_, H_5b_, H_6b_, H_6c_, m), 7.08–7.15 (H_5d_, m), 7.21 (H_2b_, d, *J* = 1.7), 7.29 (H_5d_, dd, *J* = 8.5, 1.8 Hz), 7.45 (H_3d_ , d, *J* = 2.0 Hz), 7.46 (H_2_, d, *J* = 15.5 Hz), 7.81 (H_3_, d, *J* = 15.5 Hz), 8.06 (H_2a_, H_6a_, d, *J* = 8.8 Hz); ^13^C-NMR (CDCl_3_): δ 22.45 (CH_2_), 28.67 (CH_2_), 28.73 (CH_2_), 56.02 (2OCH_3_), 67.94 (CH_2_O), 68.83 (CH_2_O), 110.12 (C_2b_), 111.15 (C_5b_), 114.27 (C_3a_, C_5a_), 114.71 (C_6c_), 117.61 (C_3c_), 119.89 (C_6b_), 121.05 (C_6d_), 122.41 (C_4c_), 122.20 (C_2_), 123.0 (C_2d_), 124.24 (C_1b_), 127.56 (C_5c_), 127.63 (C_2c_), 128.13 (C_5d_), 130.10 (C_4d_), 130.76 (C_1a_), 130.77 (C_2a_, C_6a_), 131.20 (C_3d_), 142.84 (C_3_), 144.12 (C_2c_), 149.25 (C_1c_), 151.0 (C_3b_), 151.27 (C_1d_), 152.72 (C_4b_), 162.82 (C_4a_), 188.82 (C=O); EIMS: *m/z* 641.1265 [M + H]^+^, Calcd for C_34_H_32_Cl_3_O_6_: 641.1265. Anal. Calc. for C_34_H_31_Cl_3_O_6_: C 63.61, H 4.87. Found C 61.36, H 4.84. 

*(2E)-1-[4-({8-[5-Chloro-2-(2,4-dichlorophenoxy)phenoxy]octyl}oxy)phenyl]-3-(3,4-dimethoxyphenyl) prop-2-en-1-one* (**10**). Yield 45% (0.094 mmol, 64 mg); yellow oil, IR (cm^−1^): ν_max_ 2933 (C-H), 1601 (C=O), 1512 (C=C), 1471 (C=C_Ar_), 1261 (C-O-C), 1166 ((C=O)-O), 804 (C-H_Ar_), 740 (C–Cl); ^1^H-NMR (CDCl_3_): δ 1.19–1.35 (2H, m), 1.37–1.58 (6H, m), 1.73–1.99 (4H, m), 3.46 (CH_2_O, t, *J* = 6.8 Hz), 3.98 (OCH_3_, s), 4.01 (OCH_3_, s), 4.08 (CH_2_O, t, *J* = 6.5 Hz), 6.66 (H_3c_, d, *J* = 8.8 Hz), 6.94 (H_6d_, d, *J* = 8.8 Hz), 6.97–7.05 (H_3a_, H_5a_, H_5b_, H_6b_, H_6c_, m), 7.11 (H_4c_, dd, *J* = 8.8, 2.5 Hz), 7.21 (H_2b_, d, *J* = 1.8 Hz), 7.28 (H_5d_, dd, *J* = 8.5, 1.8 Hz), 7.45 (H_3d_, d, *J* = 2.5 Hz), 7.46 (H_2_, d, *J* = 15.5 Hz), 7.81 (H_3_, d, *J* = 15.5 Hz), 8.07 (H_2a_, H_6a_, d, *J* = 8.8 Hz); ^13^C-NMR (CDCl_3_): δ 25.70 (CH_2_), 25.93 (CH_2_), 28.10 (CH_2_),28.70 (CH_2_), 32.78 (CH_2_), 34.03 (CH_2_), 56.01 (2OCH_3_), 68.21 (CH_2_O), 69.0 (CH_2_O), 110.10 (C_2b_), 111.15 (C_5b_), 114.28 (C_3a_, C_5a_), 114.62 (C_6c_), 117.62 (C_3c_), 119.9 (C_6b_), 120.85 (C_6d_), 122.37 (C_4c_), 122.20 (C_2_), 123.0 (C_2d_), 124.22 (C_1b_), 127.51 (C_5c_), 127.61 (C_2c_), 128.12 (C_5d_), 130.10 (C_4d_), 130.76 (C_1a_), 130.77 (C_2a_, C_6a_), 131.12 (C_3d_), 142.83 (C_3_), 144.10 (C_2c_), 149.24 (C_1c_), 151.1 (C_3b_), 151.26 (C_1d_), 152.72 (C_4b_), 162.93 (C_4a_), 188.81 (C=O); EIMS: *m/z* 683.1736 [M + H]^+^, Calcd for C_37_H_38_Cl_3_O_6_: 683.1734. Anal. Calc. for C_37_H_37_Cl_3_O_5_: C 64.97, H 5.45. Found C 64.07, H 6.04.

#### 3.1.4. General Procedure for the Synthesis of Triclosan-Coumarin and Triclosan-Chromone Hybrids

Triclosan (1.1 eq.), potassium hydroxide (2 eq.) and acetonitrile (10 mL), were placed in a 50 mL flat-bottomed flask equipped with a magnetic stirring bar. The mixture was stirred and heated to reflux for a period of 5 min under microwave irradiation. Then, bromoalkylcoumarin or bromoalkylchromone (1 eq.) was added to the reaction mixture which was refluxed for 30 min. The crude reaction mixture was concentrated on a rotatory evaporator, and the residue was purified by column chromatography over silica gel eluting with hexane-ethyl acetate (4:1 and then 3:2 ratio) to obtain the triclosan-chalcone hybrids in yields between 50%–80%. Monitoring of the reaction progress and product purification was carried out by TLC.

*7-{3-[5-Chloro-2-(2,4-dichlorophenoxy)phenoxy]propoxy}-4-methyl-2H-chromen-2-one* (**16**). Yield 80% (0.270 mmol, 136.8 mg); white solid, M.p. 139–141 °C; IR (cm^−1^): ν_max_ 2968 (C-H), 1718 (C=O), 1610 (C=C), 1477 (C=C_Ar_), 1263 (C-O-C), 1144 ((C=O)-O), 847 (C-H_Ar_), 740 (C–Cl); ^1^H-NMR (CDCl_3_): δ 1.14–1.25 (2H, m), 2.44 (CH_3_, s), 3.98 (CH_2_O, t, *J* = 6.6 Hz), 4.18 (CH_2_O, t, *J* = 5.8 Hz), 6.18 (H_2_, s), 6.61 (H_3b_, d, *J* = 8.8 Hz), 6.76 (H_7_, d, *J* = 2.5 Hz), 6.79 (H_5_, dd, *J* = 9.0, 2.5 Hz), 6.96 (H_6b_, d, *J* = 2.5 Hz), 6.97–7.01 (H_4b_, H_6c_, m), 7.03–7.08 (H_5c_, m), 7.43 (H_3c_, d, *J* = 2.5 Hz), 7.51 (H_4_, d, *J* = 8.5 Hz); ^13^C-NMR (CDCl_3_): δ 18.67 (CH_3_), 28.82 (CH_2_), 64.21 (CH_2_O), 65.14 (CH_2_O), 101.76 (C_7_), 112.02 (C_5_), 112.10 (C_2_), 113.75 (C_4a_), 115.0 (C_6b_), 117.51 (C_3b_), 121.43 (C_6c_), 122.40 (C_4b_), 124.26 (C_2c_), 125.55 (C_4_), 127.58 (C_5c_), 127.80 (C_5b_), 130.15 (C_4c_), 130.82 (C_3c_), 142.91 (C_2b_), 150.74 (C_1b_), 152.50 (C_1c_), 155.22 (C_7a_), 158.0 (C_3_), 161.30 (C_6_), 161.70 (C=O); EIMS: *m/z* 505.0371 [M + H]^+^, Calcd for C_25_H_20_Cl_3_O_5_: 505.0376. Anal. Calc. for C_25_H_19_Cl_3_O_5_: C 59.37, H 3.79. Found C 59.92, H 3.91.

*7-{4-[5-Chloro-2-(2,4-dichlorophenoxy)phenoxy]butoxy}-4-methyl-2H-chromen-2-one* (**17**). Yield 53% (0.170, 88 mg); white solid, M.p. 111–113 °C; IR (cm^−1^): ν_max_ 2956 (C-H), 1735 (C=O), 1625 (C=C), 1474 (C=C_Ar_), 1294 (C-O-C), 1153 ((C=O)-O), 851 (C-H_Ar_), 742 (C–Cl); ^1^H-NMR (CDCl_3_): δ 1.72–1.84 (2H, m), 1.84–1.96 (2H, m), 2.44 (CH_3_, s), 4.01 (CH_2_O, t, *J* = 6.0 Hz), 4.05 (CH_2_O, t, *J* = 5.7 Hz), 6.17 (H_2_, s), 6.69 (H_3b_, d, *J* = 8.8 Hz), 6.80 (H_7_, d, *J* = 2.4 Hz), 6.86 (H_5_, dd, *J* = 8.8, 2.4 Hz), 6.97–7.0 (H_4b_, H_6b_, m), 7.0–7.03 (H_6c_, m), 7.12 (H_5c_, dd, J = 8.9, 2.5), 7.43 (H_3c_, d, *J* = 2.5 Hz), 7.51 (H_4_, d, *J* = 8.6 Hz); ^13^C-NMR (CDCl_3_): δ 18.67 (CH_3_), 25.51 (CH_2_), 25.58 (CH_2_), 67.83 (CH_2_O), 68.62 (CH_2_O), 101.41 (C_7_), 112.0 (C_5_), 112.55 (C_2_), 113.55 (C_4a_), 114.82 (C_6b_), 118.0 (C_3b_), 121.2 (C_6c_), 122.08 (C_4b_), 124.46 (C_2c_), 125.50 (C_4_), 127.61 (C_5c_), 127.91 (C_5b_), 130.16 (C_4c_), 130.62 (C_3c_), 143.12 (C_2b_), 150.80 (C_1b_), 152.55 (C_1c_), 155.30 (C_7a_), 155.7 (C_3_), 161.35 (C_6_), 162.0 (C=O); EIMS: *m/z* 519.0536 [M + H]^+^, Calcd. for C_26_H_22_Cl_3_O_5_: 519.0533. Anal. Calc. for C_26_H_21_Cl_3_O_5_: C 60.08, H 4.07. Found C 60.02, H 4.10.

*7-({5-[5-Chloro-2-(2,4-dichlorophenoxy)phenoxy]pentyl}oxy)-4-methyl-2H-chromen-2-one* (**18**). Yield 67% (0.206 mmol, 110 mg); white solid, M.p. 84–86 °C; IR (cm^−1^): ν_max_ 2936 (C-H), 1740 (C=O), 1611 (C=C), 1469 (C=C_Ar_), 1285 (C-O-C), 1192 ((C=O)-O), 847 (C-H_Ar_), 740 (C–Cl); ^1^H-NMR (CDCl_3_): δ 1.36–1.51 (2H, m), 1.67–1.86 (4H, m), 2.43 (CH_3_, s), 3.98 (CH_2_O, t, *J* = 6.3 Hz), 3.99 (CH_2_O, t, *J* = 5.9 Hz), 6.16 (H_2_, s), 6.66 (H_3b_, d, *J* = 8.8 Hz), 6.82 (H_7_, d, *J* = 2.4 Hz), 6.87 (H_5_, dd, *J* = 8.8, 2.4 Hz), 6.93–7.06 (H_4b_, H_6b_, H_6c_, m), 7.10 (H_5c_, dd, *J* = 8.8, 2.5), 7.43 (H_3c_, d, *J* = 2.5 Hz), 7.52 (H_4_, d, *J* = 8.6 Hz); ^13^C-NMR (CDCl_3_): δ 18.70 (CH_3_), 22.45 (CH_2_), 28.61 (CH_2_), 28.67 (CH_2_), 68.3 (CH_2_O), 68.84 (CH_2_O), 101.44 (C_7_), 111.90 (C_5_), 112.60 (C_2_), 113.52 (C_4a_), 114.75 (C_6b_), 117.66 (C_3b_), 121.05 (C_6c_), 122.36 (C_4b_), 124.27 (C_2c_), 125.51 (C_4_), 127.55 (C_5c_), 127.66 (C_5b_), 130.08 (C_4c_), 130.75 (C_3c_), 143.0 (C_2b_), 151.0 (C_1b_), 152.61 (C_1c_), 152.70 (C_7a_), 155.31 (C_3_), 161.40 (C_6_), 162.11 (C=O); EIMS: *m/z* 533.0692 [M + H]^+^, Calcd. for C_27_H_24_Cl_3_O_5_: 533.0689. Anal. Calc. for C_27_H_23_Cl_3_O_5_: C 60.75, H 4.34. Found C 60.60, H 4.43.

*7-({8-[5-Chloro-2-(2,4-dichlorophenoxy)phenoxy]octyl}oxy)-4-methyl-2H-chromen-2-one* (**19**). Yield 75% (0.204 mmol, 117 mg); yellow pale oil; IR (cm^−1^): ν_max_ 2936 (C-H), 1729 (C=O), 1613 (C=C), 1474 (C=C_Ar_), 1268 (C-O-C), 1146 ((C=O)-O), 839 (C-H_Ar_), 742 (C–Cl); ^1^H-NMR (CDCl_3_): δ 1.16–1.37 (6H, m), 1.39–1.55 (2H, m), 1.59–1.72 (2H, m), 1.77–1.94 (2H, m), 2.43 (CH_3_, s), 3.94 (CH_2_O, t, *J* = 6.2 Hz), 4.05 (CH_2_O, t, *J* = 6.5 Hz), 6.16 (H_2_, s), 6.66 (H_3b_, d, *J* = 8.8 Hz), 6.84 (H_7_, d, *J* = 2.5 Hz), 6.89 (H_5_, dd, *J* = 8.8, 2.5 Hz), 6.95 (H_4b_, dd, *J* = 8.8, 2.5 Hz), 6.97–7.06 (H_6b_, H_6c_, m), 7.11 (H_5c_, dd, *J* = 8.8, 2.5), 7.44 (H_3c_, d, *J* = 2.5 Hz), 7.52 (H_4_, d, *J* = 8.8 Hz); ^13^C-NMR (CDCl_3_): δ 18.71 (CH_3_), 25.70 (CH_2_), 25.90 (CH_2_), 28.91 (CH_2_), 29.0 (CH_2_), 29.14 (CH_2_), 29.24 (CH_2_), 68.57 (CH_2_O), 68.96 (CH_2_O), 101.36 (C_7_), 111.82 (C_5_), 112.71 (C_2_), 113.43 (C_4a_), 114.62 (C_6b_), 117.62 (C_3b_), 120.85 (C_6c_), 122.36 (C_4b_), 124.21 (C_2c_), 125.51 (C_4_), 127.51 (C_5c_), 127.60 (C_5b_), 130.06 (C_4c_), 130.73 (C_3c_), 142.82 (C_2b_), 151.1 (C_1b_), 152.66 (C_1c_), 152.71 (C_7a_), 155.32 (C_3_), 161.44 (C_6_), 162.25 (C=O); EIMS: *m/z* 575.1156 [M + H]^+^, Calcd. for C_30_H_30_Cl_3_O_5_: 575.1159.

*7-{3-[5-Chloro-2-(2,4-dichlorophenoxy)phenoxy]propoxy}-4H-chromen-4-one* (**25**). Yield 79% (0.279 mmol, 137 mg); yellow pale oil; IR (^−1^): ν_max_ 2941 (C-H), 1651 (C=O), 1599 (C=C), 1496 (C=C_Ar_), 1267 (C-O-C), 1190 ((C=O)-O), 812 (C-H_Ar_), 740 (C–Cl); ^1^H-NMR (CDCl_3_): δ 2.15–2.25 (2H, m), 3.97 (CH_2_O, t, *J* = 6.0 Hz), 4.18 (CH_2_O, t, *J* = 5.8 Hz), 6.31 (H_2_, d, *J* = 6.0 Hz), 6.60 (H_3b_, d, *J* = 8.8 Hz), 6.73 (H_4_, d, *J* = 2.3 Hz), 6.91 (H_6_, dd, *J* = 9.1, 2.3 Hz), 6.95 (H_4b_, dd, *J* = 8.7, 2.6 Hz), 6.98–7.02 (H_6b_, H_6c_, m), 7.02–7.06 (H_5c_, m), 7.42 (H_3c_, d, *J* = 2.5 Hz), 7.82 (H_3_, d, *J* = 6.0 Hz), 8.12 (H_7_, d, *J* = 9.0 Hz); ^13^C-NMR (CDCl_3_): δ 28.77 (CH_2_), 64.27 (CH_2_O), 67.58 (CH_2_O), 100.86 (C_4_), 112.97 (C_6_), 114.70 (C_2_), 114.83 (C_6b_), 117.38 (C_3b_), 118.87 (C_7a_), 121.45 (C_6c_), 122.52 (C_4b_), 124.0 (C_2c_), 127.21 (C_7_), 127.59 (C_5c_), 127.75 (C_5b_), 130.12 (C_4c_), 130.9 (C_3c_), 142.75 (C_2b_), 150.72 (C_1b_), 152.53 (C_1c_), 154.91 (C_3_), 158.20 (C_4a_), 163.13 (C_5_), 177.07 (C=O); EIMS: *m/z* 513.0032 [M + Na]^+^, Calcd. for C_24_H_17_Cl_3_O_5_Na: 513.0039.

*7-{4-[5-Chloro-2-(2,4-dichlorophenoxy)phenoxy]butoxy}-4H-chromen-4-one* (**26**). Yield 72% (0.243 mmol, 122 mg); yellow solid, M.p. 75–79 °C; IR (cm^−1^): ν_max_ 2934 (C-H), 1662 (C=O), 1598 (C=C), 1471 (C=C_Ar_), 1259 (C-O-C), 1190 ((C=O)-O), 812 (C-H_Ar_), 740 (C–Cl); ^1^H-NMR (CDCl_3_): δ 1.73–1.85 (2H, m), 1.85–1.97 (2H, m), 4.03 (CH_2_O, t, *J* = 6.1 Hz), 4.05 (CH_2_O, t, *J* = 5.7 Hz), 6.32 (H_2_, d, *J* = 6.1 Hz), 6.68 (H_3b_, d, *J* = 8.8 Hz), 6.82 (H_4_, d, *J* = 2.3 Hz), 6.92–7.0 (H_6_, H_6b_, m), 7.02–7.03 (H_6c_, m), 7.12 (H_5c_, dd, *J* = 8.8, 2.5 Hz), 7.43 (H_3c_, d, *J* = 2.5 Hz), 7.81 (H_3_, d, *J* = 6.1 Hz), 8.13 (H_7_, d, *J* = 8.9 Hz); ^13^C-NMR (CDCl_3_): δ 25.53 (2CH_2_), 67.93 (CH_2_O), 68.58 (CH_2_O), 100.84 (C_4_), 112.96 (C_6_), 114.76 (C_2_), 114.83 (C_6b_), 117.91 (C_3b_), 118.72 (C_7a_), 121.19 (C_6c_), 122.11 (C_4b_), 124.41 (C_2c_), 127.19 (C_7_), 127.62 (C_5c_), 127.89 (C_5b_), 130.16 (C_4c_), 130.65 (C_3c_), 143.04 (C_2b_), 150.76 (C_1b_), 152.51 (C_1c_), 154.86 (C_3_), 158.24 (C_4a_), 163.41 (C_5_), 177.07 (C=O); EIMS: *m/z* 505.0376 [M + H]^+^, Calcd. for C_25_H_20_Cl_3_O_5_: 505.0376. Anal. Calc. for C_25_H_19_Cl_3_O_5_: C 59.37, H 3.79. Found C 59.58, H 3.79.

*7-({5-[5-Chloro-2-(2,4-dichlorophenoxy)phenoxy]pentyl}oxy)-4H-chromen-4-one* (**27**). Yield 58% (0.186 mmol, 96 mg); yellow solid, M.p. 74–77 °C; IR (cm^−1^): ν _max_ 2951 (C-H), 1649 (C=O), 1596 (C=C), 1475 (C=C_Ar_), 1268 (C-O-C), 1195 ((C=O)-O), 809 (C-H_Ar_), 740 (C–Cl); ^1^H-NMR (CDCl_3_): δ 1.35–1.51 (2H, m), 1.67–1.86 (4H, m), 3.98 (2CH_2_O, t, *J* = 6.1 Hz), 6.30 (H_2_, d, *J* = 6.3 Hz), 6.65 (H_3b_, d, *J* = 8.9 Hz), 6.84 (H_4_, d, *J* = 2.0 Hz), 6.92–7.04 (H_5c_, H_6_, H_6b_, H_6c_, m), 7.09 (H_4b_, dd, *J* = 8.7, 2.6 Hz), 7.42 (H_3c_, d, *J* = 2.4 Hz), 7.80 (H_3_, d, *J* = 6.1 Hz), 8.13 (H_7_, d, *J* = 9.0 Hz); ^13^C-NMR (CDCl_3_): δ 22.45 (CH_2_), 28.53 (CH_2_), 28.63 (CH_2_), 68.39 (CH_2_O), 68.82 (CH_2_O), 100.88 (C_4_), 112.93 (C_6_), 114.74 (C_2_), 114.83 (C_6b_), 117.60 (C_3b_), 118.70 (C_7a_), 121.10 (C_6c_), 122.41 (C_4b_), 124.22 (C_2c_), 127.15 (C_7_), 127.55 (C_5c_), 127.61 (C_5b_), 130.10 (C_4c_), 130.80 (C_3c_), 142.83 (C_2b_), 151.0 (C_1b_), 152.71 (C_1c_), 154.86 (C_3_), 158.27 (C_4a_), 163.54 (C_5_), 177.04 (C=O); EIMS: *m/z* 519.0538 [M + H]^+^, Calcd. for C_26_H_22_Cl_3_O_5_: 519.0533. Anal. Calcd. for C_26_H_21_Cl_3_O_5_: C 60.08, H 4.07. Found C 59.39, H 4.08.

*7-({8-[5-Chloro-2-(2,4-dichlorophenoxy)phenoxy]octyl}oxy)-4H-chromen-4-one* (**28**). Yield 50% (0.142 mmol, 79 mg); yellow pale oil; IR (cm^−1^): ν_max_ 2932 (C-H), 1649 (C=O), 1599 (C=C), 1496 (C=C_Ar_), 1268 (C-O-C), 1191 ((C=O)-O), 810 (C-H_Ar_), 740 (C–Cl); ^1^H-NMR (CDCl_3_): δ 1.17–1.39 (4H, m), 1.40–1.58 (4H, m), 1.60–1.75 (2H, m), 1.79–1.94 (2H, m), 3.95 (CH_2_O, t, *J* = 6.1 Hz), 4.08 (CH_2_O, t, *J* = 6.4 Hz), 6.32 (H_2_, d, *J* = 6.1 Hz), 6.66 (H_3b_, d, *J* = 8.7 Hz), 6.87 (H_4_, d, *J* = 2.0 Hz), 6.93–7.06 (H_5c_, H_6_, H_6b_, H_6c_, m), 7.11 (H_4b_, dd, *J* = 8.9, 2.4 Hz), 7.45 (H_3c_, d, *J* = 2.4 Hz), 7.81 (H_3_, d, *J* = 6.1 Hz), 8.15 (H_7_, d, *J* = 8.9 Hz).^13^C-NMR (CDCl_3_): δ 25.69 (CH_2_), 25.88 (CH_2_), 28.92 (CH_2_), 28.97 (CH_2_), 29.13 (CH_2_), 29.20 (CH_2_), 68.67 (CH_2_O), 69.0 (CH_2_O), 100.89 (C_4_), 112.95 (C_6_), 114.68 (C_2_), 114.87 (C_6b_), 117.64 (C_3b_), 118.66 (C_7a_), 120.88 (C_6c_), 122.35 (C_4b_), 124.25 (C_2c_), 127.18 (C_7_), 127.50 (C_5c_), 127.62 (C_5b_), 130.73 (C_4c_), 130.80 (C_3c_), 142.88 (C_2b_), 151.09 (C_1b_), 152.72 (C_1c_), 154.82 (C_3_), 158.29 (C_4a_), 163.71 (C_5_), 177.1 (C=O); EIMS: *m/z* 561.1003 [M + H]^+^, Calcd. for C_29_H_28_Cl_3_O_5_: 561.1002.

*Solubility*: Compounds **7**–**10**, **16**–**19** and **25**–**28** are soluble in dichloromethane, ether and ethyl acetate, which is in agreement with their low polarity. 

### 3.2. Biological Activity Assays

The compounds were subjected to *in vitro* cytotoxic activity on mammalian cells and leishmanicidal activity on intracellular amastigotes of *L. panamensis*.

#### 3.2.1. *In Vitro* Cytotoxic Activity in Mammalian Cells

The cytotoxic activity of the compounds was assessed based on the viability of the human promonocytic cell line U937 (ATCC CRL-1593.2^TM^) evaluated by the MTT (3-(4,5-dimethylthiazol-2-yl)-2,5-diphenyltetrazolium bromide) assay [41]. In brief, cells were grown in 96-well cell-culture dishes at a concentration of 100,000 cells/mL in RPMI-1640 supplemented with 10% FBS and the corresponding concentrations of the compounds, starting at 200 µg/mL in duplicate. The cells were incubated at 37 °C with 5% CO_2_ for 72 h in the presence of the compounds, and then the effect was determined using the MTT assay, incubating at 37 °C for 3 h. The effect of the compounds was determined by measuring the activity of the mitochondrial dehydrogenase by adding 10 µL/well of MTT solution (0.5 mg/mL) and incubating at 37 °C for 3 h. The reaction was stopped by adding a 50% isopropanol solution with 10% sodium dodecyl sulfate for 30 min. Cell viability was determined based on the quantity of formazan produced, according to the optical density (O.D) obtained at 570 nm in a Bio-Rad (Hercules, CA, USA) ELISA instrument. Cultured cells in the absence of extracts were used as viability controls, while meglumine antimoniate and amphotericin B were used as cytotoxicity controls. Assays were performed twice with three replicates per each concentration tested.

#### 3.2.2. Activity against Intracellular Amastigotes

The leishmanicidal activity of compounds was evaluated on intracellular amastigotes of *L. panamensis* transfected with the green fluorescent protein gene (MHOM/CO/87/UA140pIR-GFP) [42]. Effect of each compound was determined according to the inhibition of the infection evidenced by both decrease of the infected cells and decrease of intracellular parasite load. Briefly, U-937 human cells at a concentration of 3 × 10^5^ cells/mL in RPMI 1640 and 0.1 μg/mL of PMA (phorbol-12-myristate-13-acetate) were infected with promastigotes in stationary phase growth in a 15:1 parasites per cell ratio and incubated at 34 °C and 5% CO_2_ for 3 h. Cells were washed two times with phosphate buffer solution (PBS) to eliminate not internalized parasites. Fresh RPMI 1640 1 mL was added and cells were incubated during 24 h to guarantee multiplication of intracellular parasites.

After 24 h of infection, the culture medium was replaced by fresh culture medium containing each compound at concentrations of 20 μg/mL. After 72 h, the inhibition of the infection progress was determined. Cells were removed from the bottom plate with a trypsin/EDTA (250 mg) solution. Recovered cells were centrifuged at 1100 rpm during 10 min at 4 °C, the supernatant was discarded and cells were washed with 1 mL of cold PBS and centrifuged at 1100 rpm during 10 min at 4 °C. Supernatant was discarded and cells were suspended in 500 μL of PBS and analyzed by flow cytometry (FC 500MPL, Cytomics, Brea, CA, USA) counting 20.000 events. All determinations for each compound and standards were carried out in triplicate, in two isolated experiments [41,42]. Activity of tested compounds was carried out in parallel with infection progress in culture medium alone and in culture medium with amphotericin B 0.05 μg/mL and meglumine antimoniate (10.0 μg/mL) as control drugs. Compounds that showed growing inhibition percentages higher than 50% were then evaluated at four additional concentrations to determine the 50% effective concentration (EC_50_). The infected cells were exposed against each concentration of synthesized compounds during 72 h, then, cells were removed and tested by flow cytometry as described before.

#### 3.2.3. Statistical Analysis

Cytotoxicity was determined according to viability and mortality percentages obtained for each isolated experiment (compounds, amphotericin B, meglumine antimoniate and culture medium alone). The results were expressed as 50 lethal concentrations (LC_50_), that corresponds to the concentration necessary to eliminate 50% of cells, calculated by Probit analysis [45]. Percentage of viability was calculated by Equation (1), where the optical density (O.D) of control, corresponds to 100% of viability. In turn, mortality percentage corresponds to 100% viability:

% Viability = (O.D Exposed cells)/(O.D Control cells) × 100
(1)

The degree of toxicity was established according to the LC_50_ value using the following scale: highly toxic: LC_50_ < 50 μg/mL, toxic: LC_50_ > 50 to < 100 μg/mL; moderately toxic: LC_50_ > 100 to < 200 μg/mL and potentially non-toxic: LC_50_ > 200 μg/mL.

Antileishmanial activity was determined according to percentage of infected cells and parasite load obtained for each experimental condition by the cytometer. Percentage of infected cells was determined as the number of positive events by double fluorescence (green for parasites and red for cells) using dotplot analysis. On the other hand, the parasitic load was determined by analysis of mean fluorescence intensity (MFI) [41].

The parasitemia inhibition was calculated by Equation (2), where the MFI of control, corresponds to 100% of parasitemia. In turn, inhibition percentage corresponds to 100% Parasitemia. Results of antileishmanial activity was expressed as 50% effective concentrations (EC_50_) determined by the Probit method [45]:

% Parasitemia = (MFI Exposed parasites)/(MFI Control parasites) × 100(2)

The degree of leishmanicidal activity was established according to the EC_50_ value, using the following scale: Highly active: EC_50_ < 20 μg/mL, moderately active: EC_50_ > 20 to < 100 μg/mL; potentially non active: EC_50_ > 100 μg/mL.

The selectivity index (SI), was calculated by dividing the cytotoxic activity and the leishmanicidal activity using the following formula: SI = CL_50_/CE_50_. Cytotoxic compound: LC_50_ < 100 µg/mL. Non-cytotoxic compound: LC_50_ > 200 µg/mL.

## 4. Conclusions

The design, synthesis, and antileishmanial screening of twelve triclosan derivatives are reported. Several of the synthetic compounds have potential as templates for drugs development. Owing to the high leishmanicidal activity and the low cytotoxicity we consider that compounds **7**–**9** and **17** are good candidates. Studies on an animal model of leishmaniasis are needed to confirm the results observed *in vitro*. On the other hand, compounds **25**–**27** that were active against *Leishmania* parasite but toxic for mammalian cells still have potential to be considered as candidates for antileishmanial drug development. However, more studies on toxicity using other cell lines are needed to discriminate whether the toxicity shown by these compounds is against tumor or non-tumor cells. The results indicate that compounds containing small alkyl chains show better selectivity indices. Moreover, Michael acceptor moieties may modify both the leishmanicidal activity and cytotoxicity. The mechanism of action of these promising compounds also needs to be addressed.
